# Neuroplasticity to autophagy cross-talk in a therapeutic effect of physical exercises and irisin in ADHD

**DOI:** 10.3389/fnmol.2022.997054

**Published:** 2023-01-26

**Authors:** Alhasan Abdulghani, Mikayel Poghosyan, Aylin Mehren, Alexandra Philipsen, Elmira Anderzhanova

**Affiliations:** ^1^C. and O. Vogt Institute for Brain Research, Medical Faculty and University Hospital Düsseldorf, Henrich Heine University, Düsseldorf, Düsseldorf, Germany; ^2^Institute for Biology-Neurobiology, Freie University of Berlin, Berlin, Germany; ^3^Department of Psychiatry and Psychotherapy, University Hospital Bonn, Bonn, Germany

**Keywords:** physical exercises, ADHD, autophagy, BDNF, neuroplacticity, irisin

## Abstract

Adaptive neuroplasticity is a pivotal mechanism for healthy brain development and maintenance, as well as its restoration in disease- and age-associated decline. Management of mental disorders such as attention deficit hyperactivity disorder (ADHD) needs interventions stimulating adaptive neuroplasticity, beyond conventional psychopharmacological treatments. Physical exercises are proposed for the management of ADHD, and also depression and aging because of evoked brain neuroplasticity. Recent progress in understanding the mechanisms of muscle-brain cross-talk pinpoints the role of the myokine irisin in the mediation of pro-cognitive and antidepressant activity of physical exercises. In this review, we discuss how irisin, which is released in the periphery as well as derived from brain cells, may interact with the mechanisms of cellular autophagy to provide protein recycling and regulation of brain-derived neurotrophic factor (BDNF) signaling *via* glia-mediated control of BDNF maturation, and, therefore, support neuroplasticity. We propose that the neuroplasticity associated with physical exercises is mediated in part by irisin-triggered autophagy. Since the recent findings give objectives to consider autophagy-stimulating intervention as a prerequisite for successful therapy of psychiatric disorders, irisin appears as a prototypic molecule that can activate autophagy with therapeutic goals.

## Graphical abstract

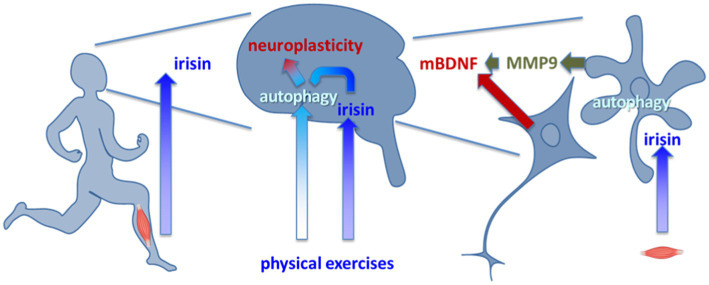

Physical activity results in irisin release. Irisin facilitates autophagy in the brain acting via glia activation. Autophagy activation favors maturation of BDNF and neuroplasticity.

## 1. Introduction

Neuroplasticity is a fundamental feature of neuronal tissue appearing as the ability of neurons to maintain and upgrade their connections and communications with respect to actual requirements (Gu and Kanai, 2014; Voss et al., 2017; Mateos-Aparicio and Rodríguez-Moreno, 2019). Neuroplasticity is a well-established mechanism of the brain to reorganize itself, both functionally and structurally and is recognized as a cause and/or accompanying trait of successful neuropsychiatric treatments. For example, the therapeutic mechanism of antidepressant activity has been shown to involve the modulation and induction of neuroplastic changes, including synaptogenesis (Castrén and Rantamäki, 2010). Changes in neuroplasticity seem to be a way to make up the neurobiological context in the brain, which could be essential to prevent precipitation, further development, or even recovery from a wide spectrum of psychiatric complications (McEwen and Chattarji, 2004; Nitsche et al., 2012).

Among the complex physiological factors that may interfere neuroplasticity, physical exercises (PEs) consistently gain attention as a supplementary or even standalone treatment of psychiatric diseases. Engagement of PEs is tempting considering their antidepressant and procognitive effects, which could involve the activation of neuroplasticity (Bettio et al., 2019; Di Liegro et al., 2019; Giménez-Meseguer et al., 2020; Swenson et al., 2020). Convenience to approach makes PEs an attractive therapeutic means, for instance in the management of depression, dementia, and attention deficit hyperactivity disorder (ADHD). However, a mechanical link between PEs and neuroplasticity is still not fully elucidated. Better understanding of molecular targets of PEs would serve the development of physiologically relevant and efficient pharmacological or genetic therapy.

One of PE’s consequences is an increase in autophagy both in peripheral organs and tissues and in the brain (Jang, 2020). Autophagy removes and helps to recycle unused macromolecules and organelles. In neurons, this fundamental function of autophagy is essential for the subcellular structural makeup required for neuroplasticity and synaptogenesis (Nikoletopoulou et al., 2017; Liang and Sigrist, 2018). Another important hallmark of PEs is the release of myokines (Jin et al., 2018). The myokine irisin initially garnered attention for its paracrine signaling leading to white to brown fat cell transformation and, upon its chronic action, to adaptation of muscle and bone tissue to increased physical load (Li H. et al., 2019; Waseem et al., 2022). However, effects of exogenous irisin (Cheng et al., 2021) or irisin gene knockdown (Islam et al., 2021) on animals’ behavior indicate that endogenous irisin stands behind activation of the muscle-brain axis and act as signaling molecule in the brain.

In the present paper, we would like to discuss the mechanism standing behind the beneficiary effects of activation of muscle-brain axis during physical activity in the frame of contemporary approaches of ADHD management and speculate if irisin signaling could be a critical component in the cross-talk between PE-induced autophagy and neuroplasticity.

## 2. The link between neuroplasticity and autophagy

### 2.1. Neuroplasticity phenomenon

Neuroplasticity is an ability of neuronal tissue to undergo functionally relevant structural and morphological changes in response to various kinds of environmental and endogenous stimuli (Gu and Kanai, 2014; Voss et al., 2017; Mateos-Aparicio and Rodríguez-Moreno, 2019). Basically, neuroplasticity mechanisms fortify functional connectivity between neurons supporting neuronal wiring. On the electrophysiological level, the phenomena of neuroplasticity can be seen as a long-term potentiation and long-term depression of neuronal activity; molecular events include changes in gene expression, post-translational modification of proteins, and changes in the activities and traffic of membrane receptors and proteins involved in signal transduction (Chen et al., 2021; Evans et al., 2021; Lee and Fields, 2021; Lim et al., 2022).

Neuroplasticity is essential for all forms of development and learning, and, therefore, in general, its high level is an adaptive feature of the brain (Compte et al., 2000; Spaak et al., 2017). In the majority of neuropsychiatric disorders, the decrease in neuroplasticity and synaptogenesis in the forebrain is often observed with no explicit nosological specificity (Goto et al., 2010; Bernardinelli et al., 2014; Vyas et al., 2016). In turn, an increase in neuroplasticity is regarded as an indicator of successful therapy across a variety of psychiatric disorders. Facilitation or strong maintenance of neuroplasticity, resulting from pharmacological interventions, is associated with an improvement of cognitive functions and top-down control of emotions (Kraus et al., 2017; Kugathasan et al., 2017). However, in a certain neurobiological context, for instance, during fear learning or development of drug dependence, neuroplasticity facilitation is maladaptive (Grimm et al., 2003; Lu et al., 2004). The region-specific increase in neuroplasticity occurs in the basal ganglia upon chronic stress and in depression (Mitra et al., 2005; Friedel et al., 2009).

Changes in neuronal wiring normally imply building up new synapses, however, they may occur without strong changes in neuronal morphology (Bhatt et al., 2009). Both ultrastructural changes and *de novo* synaptogenesis are supported by protein synthesis, reorganization of cellular milieu, and degradation of cellular components (Daskalaki et al., 2022; Kuijpers, 2022). As such, neuroplasticity implies a fine balance of ana- and cataplasticity that requires recycling and reutilization of macromolecules (Liang and Sigrist, 2018; Nikoletopoulou and Tavernarakis, 2018; Liang, 2019).

### 2.2. Autophagy as a major instrument of structural reorganization of cell

Autophagy is one of the most powerful mechanisms for maintaining protein homeostasis in the cell (He and Klionsky, 2009; Ohsumi, 2014; Anding and Baehrecke, 2017). It is a universal evolutionary conserved function, which is observed at every level of biological object complexity, from the single-cell to high-level organisms (Mizushima et al., 2004; Maday and Holzbaur, 2014; Hibshman et al., 2018). There are three types of autophagy: macroautophagy, microautophagy, and chaperone-mediated autophagy. Usually, the term autophagy refers to macroautophagy (Mijaljica et al., 2011; Kaushik and Cuervo, 2012).

Autophagy is a compartment-specific cellular milieu quality control mechanism to degrade and remove damaged organelles, misfolded proteins, or protein aggregates in a selective manner and reutilize the macromolecules after their decomposition (Mizushima and Klionsky, 2007; Naiki and Nagai, 2009; Mizushima and Komatsu, 2011; Wei et al., 2015). Thus, autophagy is protecting a cell from distress, such as starvation or chemical stress, and serving its survival during a period of restricted living conditions (Dikic, 2017; Bar-Yosef et al., 2019).

**Figure 1:**
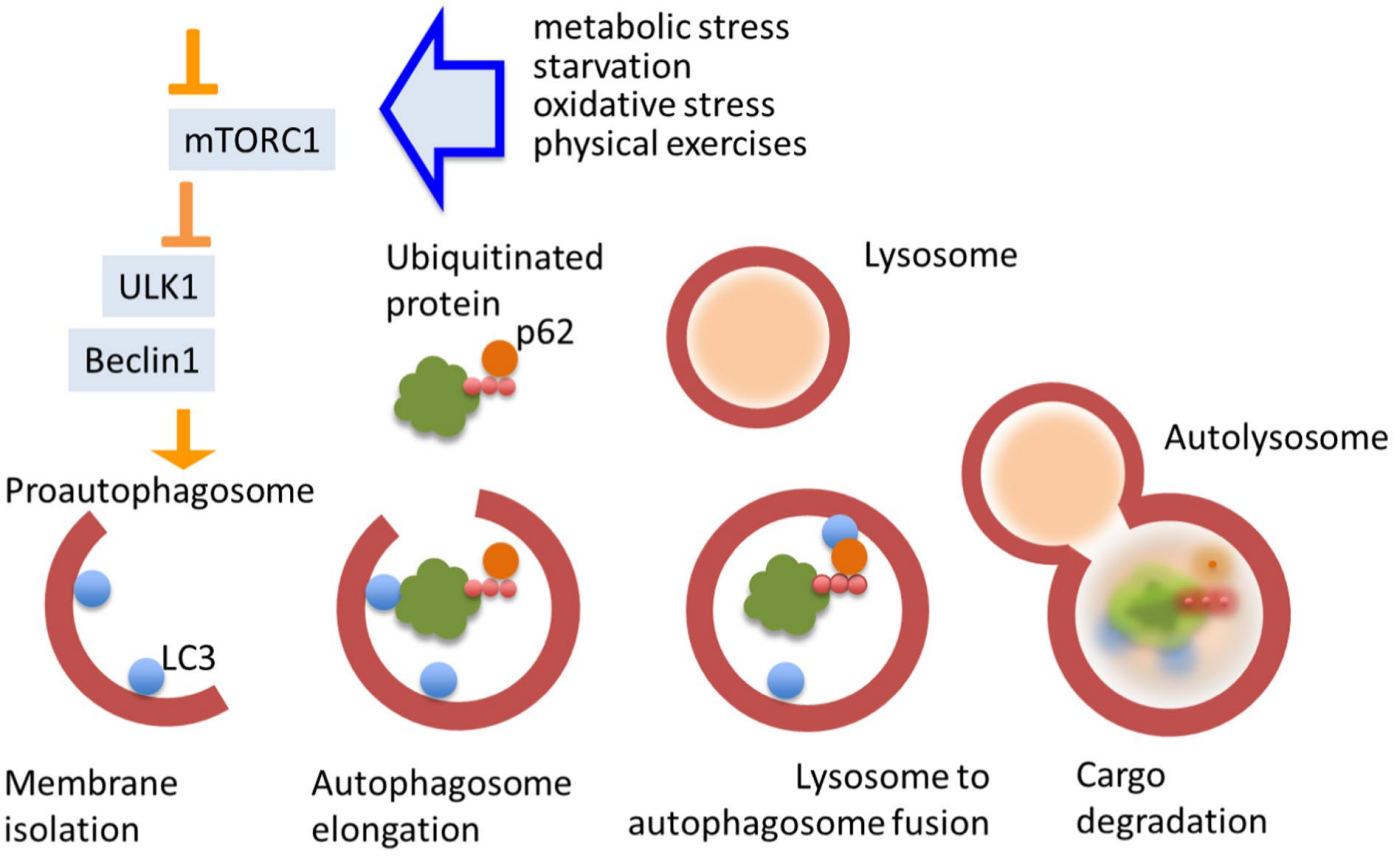
Autophagy flux. Autophagy is a pivotal mechanism of cellular homeostasis. It is triggered by chemical stress and its flux is regulated by multiple cellular signaling pathways. Autophagy strongly depends on the activity of mTORC1. Unc-51 like autophagy activating kinase (ULK1) and Becline1 are essential initiators of autophagosome formation. Cargo specificity is conferred by autophagic receptors that recognize specific target (proteins and other macromolecules), linked to p62, for degradation and microtubule-associated protein 1A/1B-light chain 3 LC3. Autolysosomes are docked to cellular membranes with subsequent devastation and release of cargo.

Autophagy is regulated by plenty of evolutionarily conserved autophagy related genes (atg) and respective regulatory proteins, for instance Unc-51 like autophagy activating kinase (ULK1) (homologue ATG1 in yeast) and Beclin1 (homologue ATG6 in yeast; He and Klionsky, 2009; Nakatogawa et al., 2009). These proteins are acting at the very early stages of proautophagosome formation and their inhibition prevents macroautophagy-dependent autophagy flux in the cell. Autophagy can also be controlled by many upstream signals in neurons. The mammalian target of rapamycin complex 1 (mTORC1) is a key suppressor of autophagy (Fukumoto et al., 2019). It supports a link between autophagy and a number of crucial intracellular signaling cascades, such as metabotropic receptor, grow factor receptor, glucocorticoid receptor signaling, as well as carbohydrate metabolism and differentiation (LiCausi and Hartman, 2018). Autophagy flux implies the formation of pro-autophagosomes, sequestration of cytoplasmic materials (autophagosome cargo) into double-membraned autophagosomes, their fusion with lysosomes or late endosomes, and degradation of cargos. The last that can be proved by a fact of sequestosome-1 degradation (also known as the ubiquitin-binding protein p62) (Mizushima and Komatsu, 2011). Cargo specificity is conferred by autophagic cargo receptors that recognize specific targets for degradation, as well as by microtubule-associated protein 1A/1B-light chain 3 (LC3), a protein that is anchored within the autophagosomal membrane (He and Klionsky, 2009). Autophagosomes are docked to cellular membranes with subsequent devastation and release of cargo into the extracellular space (Figure 1). Autophagy also coordinates with other systems of vesicular and molecular transport in the cell and with other fundamental mechanisms like apoptosis, often opposing the last one (Zhao Y. et al., 2022).

Overall, autophagy appears as a fundamental feature for providing a cell its ability to structurally reorganize and adapt.

### 2.3. Interlacement of autophagy with neuroplasticity

In neurons, autophagy influences core neuroplasticity mechanisms, both on presynaptic and postsynaptic levels. Autophagy may control vesicular release of neurotransmitters: activation of autophagy in atg7-KO mice resulted in an elevation of the evoked release of dopamine from slices of dorsal striatum (Hernandez et al., 2012). This effect may be mediated by rab26 adaptor proteins expressed on the synaptic vesicles (Binotti et al., 2015). The impairment of glutamate and gamma-aminobutyric acid (GABA)-mediated long-term plasticity in the neocortex and hippocampus was shown in mice deficient in Beclin1, the protein which is essential to initiate autophagosome formation. The changes in baseline synaptic transmission can be attributed to impairment in GABA and α-amino-3-hydroxy-5-methyl-4-isoxazolepropionic acid (AMPA) receptor trafficking and recycling due to a decrease in autophagy flux (Lalo et al., 2022). The deficits in synaptic plasticity [decrease in long-term depression in pyramidal neurons of *Cornu Ammonis 1* (CA1) area of hippocampus] were observed under pharmacological inhibition of autophagosome formation or in conditions of post-translational suppression of atg5 expression (Kallergi et al., 2022). Interestingly, activation of autophagy may be more effective in changing the neuroplasticity phenomena at conditions of pathological cellular endophenotypes related to accumulation of pathoproteins in APP/PS1 model of Alzheimer’s disease, such as cellular senescence (Wang et al., 2021).

Autophagy contributes to neuroplasticity *via* engagement of the mechanism controlling the efficiency of brain-derived neurotrophic factor (BDNF) signaling. Autophagy governs intracellular trafficking of BDNF in neurons (Kononenko et al., 2017) and may be involved in BDNF expression in microglia (Tan et al., 2018). Additional mechanisms of autophagy-regulated neuroplasticity include the regulation of extracellular levels BDNF (Martinelli et al., 2021) and other signaling proteins (Zhang et al., 2015; Kimura et al., 2017).

Autophagy activation may evoke changes in the expression of BDNF due to the concurring needs in intracellular reorganization (Xu et al., 2020). In turn, an increase in BDNF expression leads to tyrosine kinase B (TrkB)-mediated increase in the activity of mTORC1, one of the key autophagy regulatory kinases (Kim and Guan, 2015).

The type of autophagy and the time-window of its engagement may be the critical factors determining the outcome of its activation. In certain contexts, a decrease in autophagy appears as a prerequisite for cognitive function improvement (Nikoletopoulou et al., 2017; Song et al., 2017; Tian et al., 2020). Nikoletopoulou et al. showed that BDNF signaling *via* the TrkB and the phosphatidylinositol-3′ kinase (PI3K)/Akt pathway suppresses autophagy *in vivo* in the mPFC and hypothalamus. The suppression of lytic autophagy was required for structural reorganization of synapses (increase of synaptogenesis markers PSD-95, PICK1, and SHANK3 levels) and for memory enhancement under conditions of nutritional stress (Nikoletopoulou et al., 2017). The decrease in lytic autophagy is not necessarily associated with a decrease in secretory autophagy and this uncoupling may occur, for instance under stress conditions or an increase in glucocorticoid receptor signaling (Martinelli et al., 2021). As a releasing mechanism, secretory autophagy acts as an important regulator of intercellular signaling. Lysosome fusion uncoupling appears as a way to regulate the quality of released substance and facilitate the unconventional secretion of proteins and peptides (Ponpuak et al., 2015; Kimura et al., 2017; Urano et al., 2018). Our study showed that activation of autophagy is required for release of cathepsins and accumulation of BDNF in the mouse brain upon stress signaling activation (Anderzhanova et al., 2020; Martinelli et al., 2021).

To sum, the accumulated experimental data indicate that autophagy, due to a strong ability to govern effective concentration of functionally active proteins inside and outside of a cell, effectively modulates neuroplasticity in neurons.

Because of the strong interaction between autophagy and neuroplasticity, it is not surprising that changes in autophagy emerged as a pathogenetic mechanism of neurological (first of all neurodegenerative) and some psychiatric disorders (Tang et al., 2021; Filippone et al., 2022; Sainani et al., 2022). In turn, facilitation of autophagy is more and more recognized and appreciated as an end effector of therapeutic means with pro-cognitive activity (Tomoda et al., 2020). One of the diseases there activation of autophagy may be a core of therapeutic intervention is ADHD (Hess et al., 2021).

### 2.4. Autophagy as a target mechanism of pro-cognitive treatment of ADHD

Physical activity and caloric restriction are two major factors leading to the activation of autophagy in organisms (He et al., 2012; Bareja et al., 2019; Chung and Chung, 2019; Escobar et al., 2019; Kou et al., 2019). The exercise-evoked regulation of autophagy includes both increased autophagy flux as well as an increase in expression of important autophagy genes potentially resulting in enhanced autophagy capacity. Remarkable, the increase in autophagy due to PEs is not specific to the periphery and can be seen in brain tissues as well (Jang, 2020).

In addition, calorie restriction and PEs are both associated with an improvement in cognitive performance in animals and humans (Witte et al., 2009; Yang et al., 2014; Törpel et al., 2018; Hugenschmidt et al., 2019; Yegla and Foster, 2019; Grigolon et al., 2020; Horowitz et al., 2020; Wilke, 2020). PEs are specifically proposed as a therapeutic means to treat ADHD (Corona, 2018; Vysniauske et al., 2020). The involvement of autophagy-related mechanisms in the realization of the effects of PEs is deducted from the effect of pharmacologically active compounds with more specific targets. For instance, modulation of autophagy is a component of the pharmacological activity of Rg2 compound, an active substance of ginseng, a plant with well-known adaptogenic action. Rg2 is a steroid glycoside that activates autophagy in an AMPK-ULK1-dependent and mTORC1-independent manner. Most notable of all benefits of Rg2 is a clinical improvement of ADHD symptoms (Niederhofer, 2009; Ko et al., 2014). Induction of autophagy by Rg2 has other benefits such as improvement of cognitive behaviors in mouse models of Alzheimer’s disease and prevention of high-fat diet-induced insulin resistance (Fan et al., 2016; Chung et al., 2018). Besides Rg2, Rg1 also shows autophagy-dependent anti-apoptotic effects *via* engagement of the AMPK/mTORC1 pathway (Yang et al., 2018), as well as Rd, which has anti-apoptotic and mitophagy inducing functions (Liu et al., 2015, 2018; González-Burgos et al., 2017). Another component in the autophagy induction pathway is the Akt/protein kinase B signaling pathway, more specifically, its downstream segment, the Akt-GSK3β interaction. Pharmacologically- and genetically-evoked inhibition of these kinases has been shown to facilitate autophagy (Roe and Ren, 2011; Qi et al., 2012; Zhang et al., 2019b). As we have shown earlier, the Akt-GSK3β pathway in the medial prefrontal cortex turn up as a specific target of the paradoxical calming activity of amphetamine in LAB mice, which were validated as a model of ADHD (Yen et al., 2013). This suggests that amphetamine, in the case of its paradoxical calming activity, might have the capability to facilitate autophagy specifically at conditions leading to an ADHD-like behavioral endophenotype (Ren et al., 2016; Mancinelli et al., 2017).

## 3. Physical exercises as an alternative to the "easy come-easy go" psychostimulant treatment of ADHD

### 3.1. Pros and cons for conventional treatment of ADHD with psychostimulants

Attention deficit hyperactivity disorder is a highly prevalent multifaceted neurodevelopmental disorder that affects around 5% of children and 2.5% of adults worldwide (Faraone et al., 2015). The insufficient control over executive cognitive functions and vigilance (Pievsky and McGrath, 2018; Agha et al., 2020; Krieger and Amador-Campos, 2021) is translated into inattention, impulsivity, and hyperactivity, the major behavioral ADHD endophenotype. Genetic polymorphism studies suggest the involvement of changes in glutamatergic transmission, expression of dopamine D4 receptors, and dopamine transporters in the pathophysiology of ADHD (Hawi et al., 2015; Naaijen et al., 2017; Bauer et al., 2018; Faraone and Larsson, 2019; Hai et al., 2020).

To date, the ADHD treatment implies the use of psychostimulants methylphenidate and amphetamines (Arnold et al., 1972). These drugs evoke a temporal paradoxical calming effect, thereby allowing one to achieve an appropriate control over executive function, leading to (i) adaptive behavior or (ii) increasing the possibility for appropriate cognitive activity by way of improvement in attention, goal-oriented behavior, and working memory and (iii) increased top-down control over limbic brain. However, the mechanism of paradoxical effect of psychostimulants is barely well understood (Zhuang et al., 2001; Zang et al., 2005; Yen et al., 2013, 2015; Cortese et al., 2018; Miller et al., 2018; Endres et al., 2019; Huang et al., 2019). As believed, the drugs indirectly modulate activity of dopaminergic neurons with respective decrease in hypervigilance and reinforcement of reward circuit (Yildiz et al., 2011; Ziereis and Jansen, 2015; Albertson et al., 2016; Peña and Shi, 2016; Cortese et al., 2018; MacQueen et al., 2018). A long-term treatment with psychostimulant in ADHD patients leads to improvement in neurocognitive performance (Tsai et al., 2013), improved cognitive training-induced morphological plasticity and neuroplasticity, and to volumetric increase or decrease in reduction of the cortex, right caudate nucleus, and cerebellum (Hoekzema et al., 2011; Frodl and Skokauskas, 2012; Spencer et al., 2013; Rubia, 2018). Due to a relatively short half-life, most psychostimulants, also with novel formulations with slow release (Adler et al., 2017), have to be taken once or twice daily and require—at least in adulthood—a long-term treatment. Despite efficiency, the use of psychostimulants continues to raise concerns due to side effects: reduced appetite, insomnia, headache, and irritability (Schep et al., 2010; Lakhan and Kirchgessner, 2012; Kis et al., 2020), neurotoxic effects (Anderzhanova et al., 2002; Steinkellner et al., 2011), as well as tolerance and dependence (Fleckenstein et al., 2009; Merkel and Kuchibhatla, 2009; Lakhan and Kirchgessner, 2012).

### 3.2. Physical exercises are pro-neuroplastic and procognitive in ADHD

Besides beneficiary effects related to the increase in cardiac output and metabolic rate, PEs gain more attention as a supplementary or even standalone treatment in ADHD management aiming at the facilitation of neurocognitive performance (Corona, 2018; Christiansen et al., 2019; Rassovsky and Alfassi, 2019; Mehren et al., 2020; Vysniauske et al., 2020). The onset of PEs action toward moderation of the core symptoms of ADHD, inattention and hypervigilance, appears to be immediate. Importantly, PEs have essentially no side effects (Lenz, 2012). When compared to amphetamines, which lead to improvements in defined symptoms of ADHD, PEs have, in addition to symptomatic improvements, further reaching more complex positive effects on mental, physical, and emotional wellbeing. Table 1 summaries the essential features of conventional psychostimulant and PE treatment of ADHD.

**Table 1:** Comparison between two therapeutic approaches to manage ADHD.

Therapy feature	Stimulants	PEs
Onset of action	Short to medium, depending on formulation	Immediate
Efficacy	High	Intermediate
Side effects	Many, including tolerance and dependence	Minimal
Effect on neuroplasticity	Both adaptive and maladaptive	Adaptive

Physical exercises with intensities between 40 and 75% of maximal capacity are thought to have beneficial effects on memory, cognitive function, and neuroprotection (Dishman et al., 2006; Cotman et al., 2007; Mueller, 2007; Draganski and May, 2008). An increasing body of evidence supports that PEs induce remarkable functional and neuroanatomical plasticity in the mature brain, such as neurogenesis, angiogenesis, synaptic plasticity, and dendritic morphological remodeling (Bherer, 2015; Cassilhas et al., 2016; Phillips, 2017; Suarez-Manzano et al., 2018; Rassovsky and Alfassi, 2019). The hippocampus is one of the brain structures with the highest level of neuroplasticity (Bartsch and Wulff, 2015) and is also an area of neurogenesis in the sub-granular zone of the dentate gyrus. There are hypervolemic changes in the hippocampus (and also in amygdala) in kids with ADHD (Plessen et al., 2006). In addition, at least in the animal model of ADHD (spontaneously hypertensive rats), there is misbalanced glutamatergic signaling influencing norepinephrine release in the hippocampus (Howells and Russell, 2008; Shindo et al., 2022). Experimental studies in mice have demonstrated that PEs induce increases in volume and blood flow in the hippocampus (Firth et al., 2018; Tarkka et al., 2019; Broadhouse et al., 2020) and cortex (Chen et al., 2020). Interestingly, the hippocampus-specific effect may appear particularly as a consequence of moderate-to-vigorous physical activity (the level of which was proved by results of 7-day accelerometry), but not of cardiorespiratory fitness (Raichlen et al., 2019). Preclinical studies show that the effects of PEs on the brain are rather specific and attributable to changes in hippocampus function (Firth et al., 2018; Tarkka et al., 2019; Broadhouse et al., 2020). It is well known that the hippocampus is the gateway for the information that will be lately stored in cortex (Kol et al., 2020). Hippocampal neuroplasticity is an important factor of memory, navigation, and situational anxiety, which makes this brain region one of the most essential structures involved in the response to environmental changes (Morris et al., 2003; Bannerman et al., 2014). Therefore, the documented changes in the hippocampus after PEs also prove the beneficiary effect of PEs in ADHD.

Numerous studies associate the beneficiary effects of PEs with the upregulation of BDNF, an essential component of neuroplasticity (Kim et al., 2020; Müller et al., 2020; Nicolini et al., 2020; Park et al., 2020; Sugiyama et al., 2020), and with the transient increase in the availability of BDNF in the brain (Knaepen et al., 2010; Quan et al., 2020; Walsh et al., 2020). Since the mechanisms of PEs’ profitable effects may be similar to that of antidepressants (Björkholm and Monteggia, 2016; Castrén and Kojima, 2017), it is not surprising that PEs are beneficial in the improvement of regulation of emotional status (Zhang et al., 2019a; Liu et al., 2022). In addition to altering synapse wiring, PEs can induce neuroplasticity by acting on auxiliary functions that can lead to neuroplasticity, such as glial activation and angiogenesis (Morland et al., 2017; Li et al., 2021). Another hallmark of PEs is activation signaling along the muscle-brain axis due to increase in release of myokines (Jin et al., 2018).

Since the therapeutic efficacy of PEs is rather moderate, PEs are mostly suggested as a supplementary treatment (Seiffer et al., 2022). At the same time, action of PEs appears as an experimental paradigm to study interlacement between autophagy and neuroplasticity in the context of a search for pro-cognitive therapy. Elucidation of the mechanical link between PEs, autophagy, and neuroplasticity would serve to a hunt and development of more specific therapeutic approaches (Pedersen, 2019).

## 4. Irisin is linking PEs to neuroplasticity and autophagy in the brain

### 4.1. Irisin as a PEs proxy

Regular moderate-to-vigorous PEs are accompanied by changes across the whole body including cardiovascular adaptation and metabolic changes that definitely convey their beneficiary effects (Eijsvogels et al., 2016; Tofas et al., 2019; Larsen et al., 2020). Effects of PEs in the brain may also be directly attributed to muscle activation. Polypeptide myokines (cathepsin B, IL-6, decorin, BDNF, and irisin), which are released from myocytes upon their contractile activity, provide paracrine and endocrine signaling and mediate many changes across the body, including the brain (Kirk et al., 2020).

**Figure 2:**
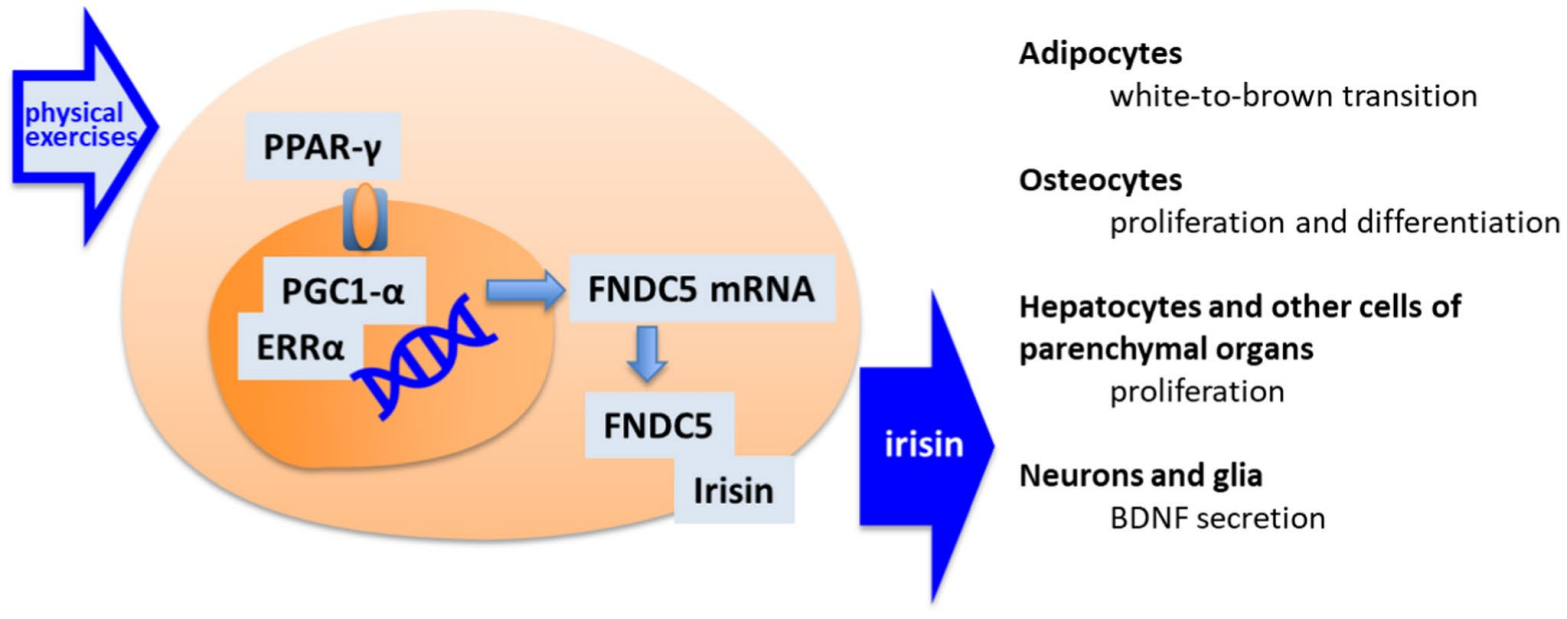
The pathway of irisin synthesis and its main cellular targets. By muscle activation, the peroxisome proliferator-activated receptor gamma (PPAR-γ) activates the PPAR receptors by giving rise to peroxisome proliferator-activated receptor gamma coactivator 1-alpha—estrogen-related receptor alpha (PGC1-α/ERRα) transcription factors for fibronectin type III domain-containing protein 5 (FNDC5) transcription. FNDC5 appears as precursor of irisin. Released Irisin accesses different organs, including brain, where it acts as an indirect stimulator of neuroplasticity.

The myokine irisin is released by cleavage of membrane fibronectin type III domain-containing protein 5 (FNDC5). The expression of FNDC5/irisin in the body is rather ubiquitous, including the brain (Dun et al., 2013), and appears under the regulation of peroxisome proliferator-activated receptor-γ coactivator 1α (PGC-1α; Lourenco et al., 2019). Expression of PGC-1α, which was initially discovered as a coactivator of mitochondrial biogenesis (Goffart and Wiesner, 2003), is upregulated by the specific activity of myocytes in skeletal muscle (Figure 2).

Irisin initially garnered particular attention for its paracrine signaling leading to white to brown fat cell transformation (Li H. et al., 2019; Waseem et al., 2022). The brown adipocytes are better serving for the metabolic challenge during physical activity by sourcing carbohydrates to provide energy to cells (Cornish et al., 2020; Kirk et al., 2020; Vidal and Stanford, 2020). Irisin and other myokines are found in central circulation in human (Di Liegro et al., 2019). The endocrine effects of irisin allows relatively slow changes in organism and synchronization of changes in the liver, pancreas, gut, and kidney, which support metabolic and detoxification needs in response to physical load (Figure 1).

We would like to highlight two mechanisms, where irisin’s peripheral action appears in relation to activation of body–brain crosstalk. It is known that, PEs are able to restore the liver homeostasis having a therapeutic effect in models of NAFLD (Seo et al., 2021; De Nardo et al., 2022). Irisin may mediate these effects supporting appropriate lipogenesis in hepatocytes, as have been shown in *in vitro* experiment with application of exogenous irisin. Changes in lipid metabolism are achieved by increase in the expression lipid-reguating enzymes LXRα and SREBP-1 and the suppression of the pathological accumulation of lipids in hepatocytes. Irisn also downregulates oxidative stress in hepatocytes as was proved by the decrease in the expression of oxidative stress markers, NFκB, COX-2, p38 MAPK, TNF, and IL-6 (Park et al., 2015). Irisin also decreases expression of enzymes involved in regulation of inflammatory response in the liver (Xu et al., 2022). Appropriate biochemical landscape in hepatocyte is a prerequisite of normal synthesis and release of hepatokines (Jensen-Cody and Potthoff, 2021; Kim et al., 2021), which, in turn, may also positively influence brain (Cooper et al., 2018; Kiyohara et al., 2021; Zhao P. et al., 2022). Another example is the positive effect of irisin on the cardiac muscle. As had been shown, irisin has effect on expression of gene with cardioprotective functions (Alzoughool et al., 2022), improves heart conditions in a model of aging in mice (Hu et al., 2022), and works as cardiomyocyte protector both *in vitro* and *in vivo* (Cao et al., 2022). An overall expected therapeutic effect is stabilization of heart contractile activity and an adequate regulation of cardiac output. In turn, the stable system hemodynamic, which depends on heart activity, is essential to maintain all the brain functions, including supplementation of neuroplastic processes.

The proof of the relevance of irisin plasma levels to the effects of PE was particularly challenging, due to a lack of specific antibodies (Albrecht et al., 2015). However, recent studies using either LC–MS measurements of irisin in plasma (Jedrychowski et al., 2015) or applying extended validation of western blot measurements of plasma irisin (Löffler et al., 2015) proved the presence of free irisin in human blood and confirmed the positive correlation between PEs and circulating irisin in human (Tsuchiya et al., 2014; Jedrychowski et al., 2015; Ruan et al., 2018; Tari et al., 2019; Qiu et al., 2020). Preclinical studies also show the coincidence of physical activity and elevation in the plasma irisin levels in healthy subjects (Murao et al., 2019). Permanent PEs accomplished as daily swimming restore the impaired levels of FNDC5/irisin in blood in the animal’s models of Alzheimer’s disease (Lourenco et al., 2019). However, there were some concerns (Albrecht et al., 2015) on the reliability of irisin as an indicator or/and mediator of the effects of PEs because of reports of reductions of irisin plasma levels in association with PEs. For instance, an acute PE resulted in an elevation of plasma irisin, while 12-week training was associated with a decrease in plasma irisin (Norheim et al., 2014).

### 4.2. Brain-specific effects of irisin and its molecular targets in the brain

The brain emerges as an important target of irisin. It seems feasible that irisin is implemented in the realization of the mechanical link between exercises and changes in the neuronal cells leading, in analogy to peripheral effects, to adaptive adjustment of lipid metabolism, cellular excitation, and antioxidant systems in neurons and glia (Stranahan et al., 2010). Effects of exogenous irisin (Cheng et al., 2021) or irisin gene knockdown (Islam et al., 2021) on animals’ behavior indicate that it also provides a functionally relevant signaling in the brain.

Irisin is found in cerebrospinal fluid (CSF) in both humans and rats (Piya et al., 2014; Ruan et al., 2019). Irisin is a glycosylated protein, and, therefore, irisin released from myocytes may penetrate the blood–brain barrier (BBB; Otvos and Wade, 2014; Islam et al., 2021) and contribute to the brain/CSF pool of irisin. However, the neuronal tissue is another source of irisin. In the brain of mice, FNSC5/irisin expression is highest in the regions related to the regulation of locomotor activity, like the cerebellum, caudate, and putamen. It is also observed in the olfactory bulbs, ventral thalamus, medial vestibular nucleus, and CA1 region of the hippocampus (Dun et al., 2013; BrainStars, 2020). Interestingly, CSF and plasma irisin levels, each independently correlate with age, Alzheimer’s disease, and obesity (Lourenco et al., 2019; Ruan et al., 2019, 2020). These data suggest that FNDC5/irisin expression in the brain and peripheral organs are regulated separately, and the sizes of peripheral and central pools of irisin could be considered as independent measures.

The factors that influence the availability of brain-derived soluble irisin in the brain are generally not known. Experimental studies are showing interlacement of mechanisms regulating the FNDC5/irisin system and controlling behavioral responses. Chronic unpredictable stress leading to increased immobility correlates with the decrease in FNDC5/irisin expression in the brain (Wang and Pan, 2016). This observation suggests the involvement of glucocorticoid receptors in the regulation of FNDC5/irisin expression (Delezie and Handschin, 2018). Also, BDNF, which is an essential neurotrophin associated with neuroplasticity, can regulate FNDC5 levels by activation of negative feedback and, therefore, can reduce the *bdnf* gene’s expression (Wrann et al., 2013). The antidepressant effect of PEs was accompanied by an increase in neuron proliferation, differentiation, and survival and associated with an increase in the number of FNDC5-positive cells in the hippocampus (Siteneski et al., 2020).

The significance of irisin signaling for brain functions is supported by experimental studies. FNDC5/irisin expression is required to maintain the normal transcriptome of newborn neurons in the hippocampus. The genes with abnormal transcription were annotated to clusters associated with stem cell and neuron differentiation, neurotrophic and insulin signaling pathways, dendrite development, as well as neurodegenerative disorders (Islam et al., 2021). Irisin microinjected in the dental gyrus of the hippocampus of Wistar rats induced long-term potentiation and facilitated lipid peroxidation. Downregulation of FNDC5/irisin by shRNA impaired synaptic plasticity and memory in mice. This phenomenon corresponded to a decrease in field ePSPs in *in vitro* preparations from the brains (Mohammadi et al., 2019). As had been shown recently, the intravenous administration of irisin in spontaneously hypertensive rats, a model of ADHD (Botanas et al., 2016), resulted in normalization of blood pressure (Huo et al., 2020) that, together with changes in the paraventricular nucleus, suggest changes in ACHT-vasopressin system. The bilateral intra-hippocampal administration of irisin diminished the negative consequences of restraint stress in mice (Jodeiri Farshbaf et al., 2020). Notably, in this study, irisin showed gender-specific effects counteracting both neurobehavioral disturbances, like anxiety and memory impairment, and changes in vegetative system regulation in males, while only preventing the reduction in body weight in females (Jodeiri Farshbaf et al., 2020). Overexpression of irisin in the hippocampus (local injection of AAV8-irisin-FLAG) improved context-dependent memory in the paradigm of Pavlovian fear conditioning (Islam et al., 2021). Wang and Pan showed, that subcutaneous administration of irisin showed a dose-dependent improvement of behavior in a model of chronic unpredictable stress counteracting the depression-like endophenotype (Wang and Pan, 2016).

Post-translational modification of FNDC5 expression with siRNA resulted in a decrease of irisin but also in a decrease in the level of UCP2 (uncoupled protein 2 serving as inner mitochondrial membrane transporter; Pan et al., 2021), therefore, indicating possible changes in production of reactive oxygen species. Exogenous irisin showed a neuroprotective effect in models of neurodegenerative disorders (Lourenco et al., 2019; Zarbakhsh et al., 2019). Local administration of recombinant irisin, as well as expression of FNDC5-containing adenovirus vector in AβO-treated mice, restored memory in the mouse model of Alzheimer’s disease. Both positive effects corresponded to the normalization of diminished field ePSPs (Lourenco et al., 2019) in the experimental mice. An elegant approach to boost the pool in irisin in the periphery used an AAV vector-associated overexpression of FNDC5/irisin in the liver. The resulting increase in the availability of peripheral irisin improved the cognitive performance of mice in two transgenic models of Alzheimer’s disease (APP/PS1 and 5xFAD), which was also accompanied with reduced glia activation (Islam et al., 2021).

A study by Boström’s group showed an interaction between FNDC5 and BDNF systems, providing evidence for the influence of endurance PEs on PGC-1α/FNDC5-dependent expression of BDNF both in the peripheral organs and in the hippocampus of mice (Wrann et al., 2013). It is worth mentioning that the increase in FNDC5—BDNF system activity in the periphery is barely adding to the changes in brain BDNF immunoreactivity, because BDNF practically does not penetrate the BBB under healthy physiological conditions (Hernandez et al., 2022). A recent study showed that subcutaneous administration of irisin resulted in an increase of dendrite complexity in the CA1 and CA3 areas of the hippocampus in coincidence with upregulation of mRNA for PGC-1α, FNDC5, and BDNF, therefore, supporting the neurotrophic mechanism of irisin action (Mongrédien et al., 2019).

Results of *in vitro* experiments suggested that brain activity of irisin may be mediated by glial cells (Wang and Pan, 2016; Lourenco et al., 2019). The discovery of receptors specific to irisin, the integrin family receptor α1β1, and especially, αvβ5 (Kim et al., 2018), strongly confirmed the involvement of astrocytes, since these cells are known to express specifically integrin receptor αvβ5 (Milner et al., 1999). Subcutaneous irisin led to upregulation of mRNA for the astrocyte marker hevin (Mongrédien et al., 2019) and downregulation of tumor growth factor 1 (TGF-β1) mRNA, which would promote neuroplasticity due to respective changes in the release of these astrocyte signaling molecules (Tewari and Majumdar, 2012; Ota et al., 2013; Kim and Leem, 2019). All these remarkable discoveries give a new impulse to study the effects of irisin in the brain using glia as a model of irisin action.

However, despite the recent promising finding showing both the existence of integrin receptor αvβ5 for irisin and its effect on glial and neuronal activity and behavior, a mechanistic link between an increase in irisin content in brain and changes in brain function is still missing.

## 5. Autophagy as targets of irisin

**Figure 3:**
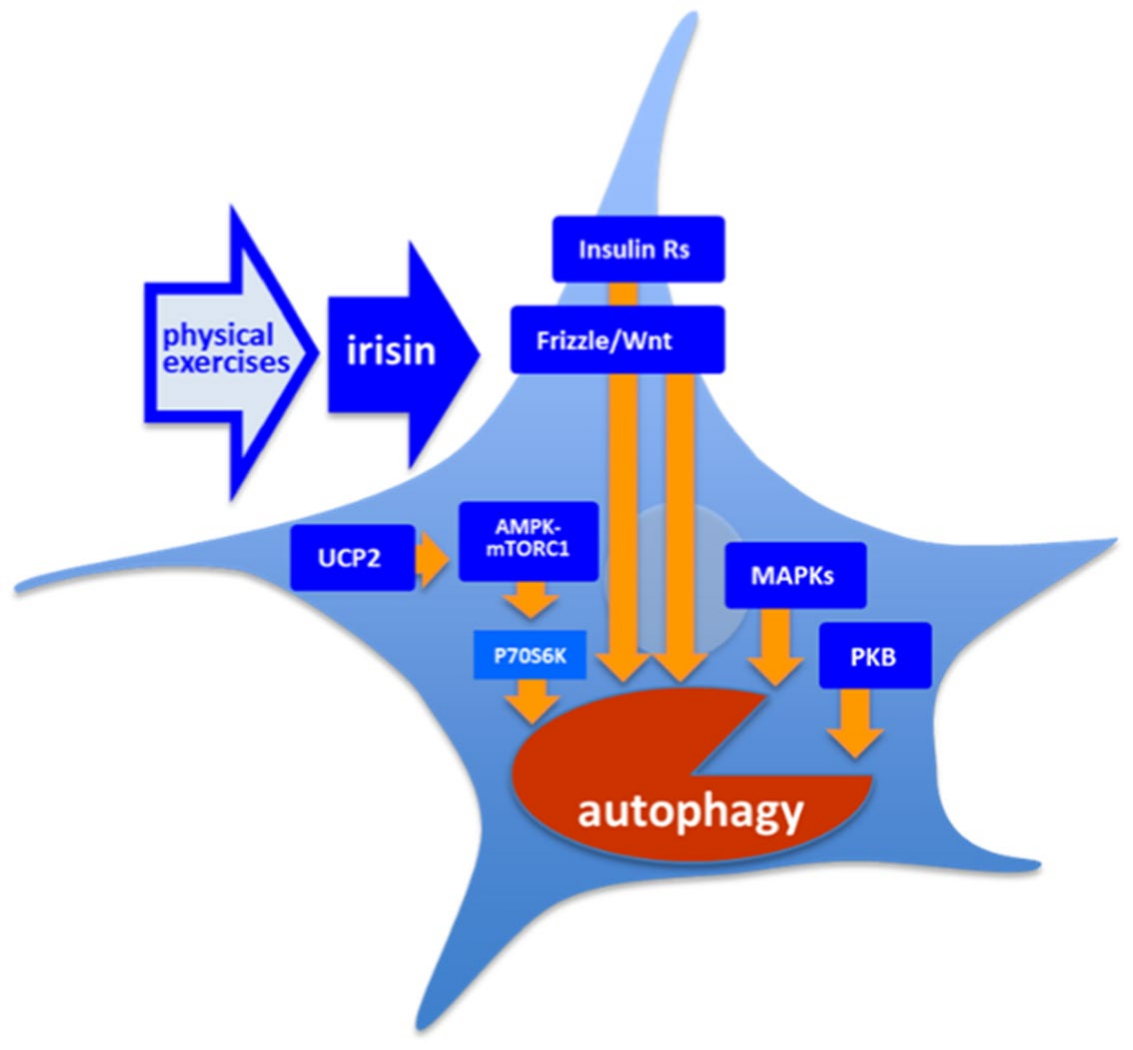
Molecular targets of irisin. There is a number of the intracellular cascades are shown to respond to irisin both *in vitro* and *in vivo*: However, the exact mechanism providing a coupling of free irisin and its intracellular targets is still unknown. AMPK, 5’ AMP-activated protein kinase; Frizzle/Wnt, key proteins of respective pathway; Insulin Rs, Trk insulin Receptors; mTORC1, mammalian target of rapamycin complex 1; PKB, protein kinase B (Akt); and UCP2, uncoupled protein 2.

Until present, the data on the molecular interaction underlying effect of irisin on autophagy are still very scattered. Analysis of the most validated intracellular targets of irisin reveals a few molecular nodes, at which the increase in irisin signaling influences autophagy. Experimental data prove such an interaction in cells of a non-neuronal lineage. Nonetheless, the respective intracellular pathways are also of a high importance in all cells and their engagement might predict the effect of irisin on autophagy in neurons and glia (Figure 3). Despite the involvement of established negative regulators, the available literature mainly reports an autophagy-activating effect of irisin, implying the existence of more active positive regulators such as mitogen-activated protein kinases (MAPKs). In astrocytes, an engagement of secondary messenger systems could be mediated by integrin receptor αvβ5. As had been shown, αvβ5 are involved in control of atg7-dependent autophagy in pulmonary endothelial cells (Zhang et al., 2019b).

Irisin leads to an increase in the activity of protein kinase B (PKB or Akt) in cardiomyoblasts, H9c2 cells, and in endothelial cells and, respectively, to an increase in activity of its downstream mTORC1 and supression of autophagy (Yang et al., 2013; Xie et al., 2015; Fu et al., 2016; Gao et al., 2021; Zajicek et al., 2022). Song et al. showed that exogenous irisin treatment (50–200 ng/ml, 24–48 h) decreased autophagy in H9c2 cells as it was seen by the decrease in LC3II/LC3I ratio. However, the authors showed that this negative effect depends in part on the PI3K/Akt signaling pathway (Song et al., 2021). As had been shown in cultured INS-1 cells, irisin activated 5’ AMP-activated protein kinase (AMPK)-mTORC1 cascade increasing survival of insulin producing cell (Lee et al., 2015; Li et al., 2018; Moon and Park, 2020). It has been shown that besides activation of AMPK-mTOR pathway, irisin led to mTORC1-independent activation of AMPK-ULK1 pathway, with both alleviating cardiac hypertrophy (Li et al., 2018; Yu et al., 2019). AMPK is also upregulated by uncoupled protein 2 (UCP2; Mao et al., 2021), which was shown to be decreased in absence of irisin in endothelial cells (Pan et al., 2021). Another pathway, which was highlighted with respect to irisin activity, is the AMPK/SIRT1/PGC-1α signaling cascade, however, this pathway was rather considered as an upstream regulator of FNDC5/irisin pathway (Li Q. et al., 2019). In addition, the Frizzle/Wnt pathway is also known to be modulated in presence of irisin, and in turn, to regulate autophagy flux in adipocytes (Petherick et al., 2013; Ma et al., 2019). The MAPKs-dependent pathway contributing to regulation of autophagy is also modulated by irisin (Mazur-Bialy et al., 2017; Li and Halterman, 2021). Worth mentioning, PEs are known to positively modulate Frizzle/Wnt signaling and MAPKs-signaling, as well as insulin signaling (Stranahan et al., 2010), which is, in turn, coupled to phosphatidylinositol 3-phosphate (PI3P)/PKB and MAPK pathways. Irisin (250 μg/kg, single dose) improves autophagy in aged hepatocytes *in vivo via* increasing telomerase activity. Moreover, the single administration of irisin results in increase in LC3II/LC3I ratio but also in the decrease in p62 expression, which may point to changes in the balance between secretory and lytic autophagy (Bi et al., 2020). Pang et al. showed that exogenous irisin (100 ng/ml, 72 h) leads to an increase in the levels of LC3II and p62, as well as to an increase to LC3II/LC3I ratio in vascular smooth muscle cells in mice. Notably, this effect goes along to the increase in levels of mRNAs of Map1lc3b, Becn1, Atg7, Atg5, and Lamp1, further highlighting epigenomic effects of irisin (Pang et al., 2022). Pan et al. showed that exogenous irisin (20 nM, 48 h) was diminishing the toxic effect of doxorubicin in the culture of endothelial cells. While irisin did not show effects when administered alone, this action was dependent on changes in autophagy flux, since cytoprotective combination of irisin and doxorubicin resulted in the prominent increase in p62 and a very moderate increase in LC3II/LC3I ratio (Pan et al., 2021). Irisin also activated Opa1-induced mitophagy to restore mitochondrial energy metabolism in cardiomyocytes (Xin and Lu, 2020).

The ability of irisin to activate the essential ubiquitous secondary messenger pathways, which are associated with regulation of autophagy, suggests a possibility for irisin to modulate autophagy in neuronal and glial cells.

### 5.1. Hypothesis: Irisin-evokes neuroplasticity by triggering autophagy

We propose that autophagy activation is a critical component of irisin action in the brain. Modulation of autophagy does not only support unspecific remodeling of cellular content but also evokes neuroplasticity. Our recent studies using FKBP51-KO mice as a model for impaired stress response give an idea of how irisin-stimulated autophagy leads to activation of neuroplasticity cascades, particularly to those required for BDNF signaling.

It is established that the stress-responsive co-chaperone FKBP51 (Binder, 2009; Touma et al., 2011) is required for autophagy-dependent antidepressant activity of amitriptyline and paroxetine (Gassen et al., 2014, 2015). Recently we have shown that FKBP51 expression in mice has a permissive role in acute ketamine antidepressant action. Moreover, FKBP51 expression is critical for the almost immediate increase in the extracellular mature BDNF (mBDNF) content induced by a single dose of ketamine (Anderzhanova et al., 2020) or acute stress (Martinelli et al., 2021). Moreover, the diminution of stress-evoked increase in extracellular mBDNF upon suppression of autophagy with ULK1 inhibitor was coincided with a decrease in release of autophagy cargo cathepsin D and matrix metalloproteinase 9 (MMP9). Analysis of the secretome of cultured astrocytes revealed that *atg5*-expression-dependent autophagy is critical for the release of MMP9. Furthermore, the *in vivo* secretome analysis showed that FKBP51-dependent increase in extracellular levels of mBDNF coincided with release of MMP9, but not pro-BDNF. Therefore, our data show that autophagy is a key for maturation of BDNF due to control of MMP9 secretion. As it appears, this mechanism is not constitutively active but is engaged on demand, during acute activation of stress-associated glucocorticoid receptor signaling or during acute action of the antidepressant ketamine.

We believe that irisin acts at astrocyte αvβ5 and, therefore, interferes with autophagy flux, and facilitates secretion of MMP9. At the same time, the activity-dependent release of pro-BDNF supplies the extracellular milieu with the MMP9 substrate. In sum, a stimulation of BDNF signaling becomes feasible in the activated structures, where irisin is expressed or perceived. The proposed scenario is taking into consideration that the overall effect of irisin depends on the pattern of αvβ5 integrin’s’ expression, and therefore its experimental proof requires discrimination of the effect of irisin in neurons and glial cells.

The proposed hypothesis, however, allows one to escape a question on the ultimate requirement of irisin upregulation in the neurons to upregulate BDNF expression since it relays only on an increase in irisin content, regardless of the source of such an increase. Secondly, the assumable mechanism incorporates astrocyte and neuron cross-talk in the implementation of neuroplasticity. Thirdly, the hypothesis integrates autophagy and neuroplasticity into one biologically relevant process of on-demand response of the brain to changes.

## 6. Conclusion

Active movement and physical activity are emerging not only as a part of a healthy lifestyle but also as an important therapeutic means to improve brain functioning. The mechanisms of PEs therapeutic activity were engaged in human natural history. At early stages of its evolution, *Homo sapiens* was adapting to nomad life in savannah. The new circumstances required a lot of running, goal-oriented behavior, high ability for spatial navigation, and good memory together with effectuation of coordinated activity in social groups for effective foraging and defense of a tribe as a whole. This adaptation was supported by respective neuroplastic and morphological changes in the brain (Lago et al., 2019; Duckworth et al., 2020), which were stabilized in the genome (Noakes and Spedding, 2012).

Beside supporting an evolutional process in the past, mechanisms which underly effects of PEs in the brain may be a paradigm to look for a novel treatment of psychopathological complications, for instance in ADHD management. In this review, we justified and proposed that PE-related neuroplasticity is mediated in part by irisin-triggered autophagy. Since the recent findings give the objectives to consider autophagy-stimulating therapy as a prerequisite for successful therapy of psychiatric disorders, irisin appears as a prototypic molecule that can activate autophagy with therapeutic goals. There is a decisive evidence gap in the knowledge of the mechanisms of action of irisin in the brain. Future study on a mechanical link between irisin and autophagy would improve our understanding of the mechanisms involved in physiologically relevant therapy of psychiatric disorders.

## Author contributions

AA: original draft preparation, reviewing, and editing. MP and AM: writing and reviewing. AP: conceptualization and reviewing. EA: supervision, conceptualization, reviewing, and editing. All authors contributed to the article and approved the submitted version.

## Funding

Publication fees funded by Klinik und Poliklinik für Psychiatrie und Psychotherapie Universitätsklinikums Bonn Venusberg-Campus 1 Gebäude 80/82 53127 Bonn to AP.

## Conflict of interest

The authors declare that the research was conducted in the absence of any commercial or financial relationships that could be construed as a potential conflict of interest.

## Publisher’s note

All claims expressed in this article are solely those of the authors and do not necessarily represent those of their affiliated organizations, or those of the publisher, the editors and the reviewers. Any product that may be evaluated in this article, or claim that may be made by its manufacturer, is not guaranteed or endorsed by the publisher.
